# Body awareness and chronic low back pain: validity and reliability study of Turkish version of body awareness rating scale

**DOI:** 10.3906/sag-1911-110

**Published:** 2020-06-23

**Authors:** Aynur DEMİREL, Dilara ONAN, Yasemin ÖZEL ASLIYÜCE, Müzeyyen ÖZ, Utku BERBEROĞLU, Özlem ÜLGER

**Affiliations:** 1 Faculty of Physiotherapy and Rehabilitation, Hacettepe University, Ankara Turkey

**Keywords:** Low back pain, chronic pain, body image, reproducibility of results

## Abstract

**Background/aim:**

Previous studies reported that patients with chronic low back pain (CLBP) had trouble describing senses or body functions. A questionnaire, the body awareness rating questionnaire (BARQ), was recently developed for assessing body awareness. The aim of the study was to develop a Turkish version of the BARQ and investigate the validity and reliability in patients with CLBP.

**Materials and methods:**

BARQ translated to Turkish with forward-backward method. Ninety-nine patients with CLBP and 101 healthy controls (HC) completed the BARQ-T. Fifty-one of patients with CLBP and HC repeated BARQ-T 3 days later. In addition to BARQ-T, Oswestry disability index (ODI), pain severity, short form 36 (SF-36) and Toronto alexithymia scale (TAS) were administered.

**Results:**

The current study found good-excellent Cronbach’s alpha values for patients with CLBP (α: between 0.883–0.967) and acceptable-good Cronbach’s alpha values for HC (α: between 0.649–0.825) in factors of BARQ-T. ICC values for test-retest validity were found to be good-excellent for patients with CLBP in all factors. BARQ-T was positively correlated with SF-36 and negatively correlated with ODI and TAS (P < 0.05).

**Conclusion:**

The study confirmed that the BARQ-T has acceptable validation and reliability in terms of pain perception and pain assessment in the Turkish CLBP community.

## 1. Introduction

Body awareness was known as capacity of differentiating body parts and had an important role in promoting fine coordinated movements. To achieve correct and accurate body awareness depends on the integration the afferent proprioception from joints and muscles. Body awareness copes with problems by identifying the changes in their bodies and experiencing and accepting the changes [1,2]. Some researchers reported that there is a dissonance between accurate and estimated proprioception in patients with chronic pain [3–5].

In this perspective, it is impossible to think that there are no deviations in body awareness in patients with chronic pain as to healthy people. Hence, body awareness was applied either directly or indirectly in most of the treatments for pain management. Problems for describing the senses or body functions were reported in patients who had muscle strains or tightness and long-lasting pain. Chronic low back pain (CLBP) a disease which is frequently relapsed, causes workforce loss and the treatment takes time [6]. Therefore, assessments of patients with chronic low back pain should include complementary and whole body treatments [7]. Low back pain (LBP) is a common problem affecting 80% of Turkish society, as well as a public health concern among musculoskeletal disorders. Holistic approaches, such as body awareness therapies, yoga, pilates, tai chi, and other philosophies for recognizing the body were recently becoming more popular [8–11]. Individually, all approaches are intended for self-recognition, self-realization, and self-management. Tove et al. developed a self-report questionnaire, which had 24 items including mood, feelings, function, and awareness factors that determine the effectiveness of treatments from baseline levels of body awareness [12]. This questionnaire was also used to discriminate the patients with psychosomatic disorders from musculoskeletal disorders and healthy people. Although there are many versions of this questionnaire, there was no Turkish version for native Turkish individuals to determine the body awareness ratio. Therefore, the aim of the present study was to develop a Turkish version of the body awareness rating questionnaire (BARQ-T) and investigate the validity and reliability in patients with chronic low back pain.

## 2. Materials and methods

Translation of the BARQ into Turkish was conducted using a forward-backward method by Beaton et al. After needed permission for translating the BARQ was received from Tove et al. who developed the BARQ, 3 native Turkish speakers (AD, DO, OU) translated the original BARQ items English to Turkish. A single Turkish translation was created from these 3 translations. One person who is native English speaker and has not familiar with BARQ and also speaks Turkish fluently translated this Turkish questionnaire back to English. The English questionnaire was compared with the original version, discrepancies were resolved by discussion and a provisional BARQ-T was created. Finally, this provisional BARQ-T was applied to 10 native Turkish speaker patients with CLBP. The feedback on comprehensibility and completeness of the BARQ-T were assessed as “yes, it is difficult” or “no, it is not difficult”. According to patients’ feedback, a final version of BARQ-T was developed.

### 2.1. Research ethical approval and participants

Necessary authorization and permits for this study have been secured from the Non-Entrepreneurial Clinical Studies Ethical Board of Hacettepe University. All procedures were conducted according to the Declaration of Helsinki. All participants provided informed consent. This prospective study included 99 patients with CLBP who referred to the physiotherapy unit and 101 healthy controls who were the patients’ caregivers.

CLBP participants met the following eligibility criteria: the presence of pain or symptoms higher than 3 months, aged between 18–65 years, and had pain over lumbar region and gluteal side. CLBP participants were excluded if they had any of the following: a history of spinal surgery, known spinal pathology (i.e. scoliosis, metastatic carcinoma of the spine, spondylolisthesis), radiculopathy, using antidepressive medication and/or motor and sensorial deficit which causes bladder and sexual dysfunction.

### 2.2. Procedure

The demographic data of all patients (sex, age and body mass index) and pain severity and duration were recorded. The pain severity during activity of all CLBP patients was evaluated with a visual analog scale (VAS), by the participant marking a vertical line on a 10 cm horizontal line to represent the severity of the pain where 0 = no pain, and 10 = intolerable pain.

Because of the original BARQ was used Oswestry disability index (ODI) for functional disability, short form 36 (SF-36) for quality of life and Toronto alexithymia scale (TAS) for alexithymia to indicate the construct validity, these scales were also used with BARQ-T. Hence, it has been known that patients had chronic pain conditions had difficulty for describing feelings, emotions and bodily function alexithymia was used [12,13]. 

Fifty-one of patients with CLBP and HC who participated in the study were randomly selected for test-retest reliability analysis and BARQ-T was repeated 3 days later.

BARQ: The original version of this self-reported questionnaire composed of 4 different factors that indicates the different aspects of body awareness. The factors are evaluating body awareness in terms of function, feeling, mood and awareness. Each item scores a 7-point Likert type scale. Each factor’s scores range between 6 to 42 and higher scores indicates higher body awareness [12]. 

Pain severity: The pain severity was rated by patients using a visual analog scale (VAS), a fixed 10 cm horizontal line oriented Left (“no pain”) to right (“unbearable pain”). The ends of the line indicate the extreme limits of pain severity. The distance between the point marked by the patient and the baseline (“no pain”) is measured in centimeters.

Oswestry disability index (ODI): Low back pain related disability levels were assessed with the Turkish version of the Oswestry disability index (ODI). This self-administered, reliable, and valid 10-item index was applied to patients with CLBP. ODI total scores range from zero (no disability) to 100 (severe disability) points. ODI has five values that indicate the disability level. Zero to 20% indicates a minimal disability, 20% to 40% indicates a moderate disability, 40% to 60% indicates a severe disability, 60% to 80% indicates crippled, and 80% to 100% indicates bedbound (or exaggerating symptoms) [14, 15].

Short form 36 (SF-36): Turkish version of SF-36 was used to measure the changes in quality of life (QoL) levels due to chronic low back pain. This scale consists of 36 items and assesses various subparameters such as physical function, physical role difficulty, pain, general health, energy, social function, emotional role difficulty, mental health, etc. Each subparameter is scored on a scale of 0 to 100, where 0 is the lowest and 100 is the highest score [16, 17].

Toronto alexithymia scale (TAS): Turkish version of TAS was used to measure the changes of Alexithymia levels of patients. Alexithymia has been known as having trouble for identifying and describing emotions. This self-report scale consists of 20 items and 3 subscales such as difficulty describing feelings, difficulty identifying feelings, and externally-oriented thinking. Items are rated using a 5-point Likert scale whereby from strongly disagree to strongly agree. The TAS score is the sum of responses to all 20 items, equal to or less than 51 points indicate nonalexithymia; scores between 52 to 60 points indicate possible alexithymia; equal to or greater than 61 indicates alexithymia [18,19].

### 2.3. Statistical analysis

The IBM SPSS statistical 21.0 software was used for the statistical analyses. Descriptive analyses were presented using means, standard deviations and percentages. Construct validity of the BARQ-T was determined by assessing correlations between ODI, SF-36 and TAS. According to the Spearman’s correlation coefficient (r), the relevance levels of the correlations were accepted as r ≥ 0.70 means strong, 0.30–0.70 moderate, r < 0.30 weak correlation. The internal consistency reliability and test-retest reliability were evaluated. The internal consistency reliability of each subscale measured using Cronbach’s alpha. When the alpha value is higher than 0.70, it indicates acceptable internal consistency. Intraclass correlation coefficient (ICC) value with 95% confidence intervals (CI) was calculated for test-retest reliability. ICC value is higher than 0.70 indicates acceptable test-retest reliability. BARQ-T’s factors scores of HC and patients with CLBP were calculated and receiver operating characteristic (ROC) curve analysis applied for each factor. While evaluating the area under the curve (AUC) a 5% type-1 error level was used to accept a statistically significant predictive value of the test variables [20]. An AUC of 0.70 and over is considered acceptable and higher level of AUC shows greater discriminative ability of patients with or without the disease in general [21].

## 3. Results

### 3.1. Participants

This study included 99 patients with CLBP and 101 HC between the ages of 18 and 65 years. There was no significant difference in terms of BMI, but the HC were younger than the CLBP patients (P < 0.05). The patients with CLBP had moderate level pain severity and disability, possible alexithymia, and moderate level QoL (Table 1).

**Table 1:** Descriptive statistics of participants.

Variables	CLBP(N = 99)N (%), X ± SD	HC(N = 101)N (%), X ± SD	P
Age (Years)	44.91 ± 12.92	36.94 ± 11.98	0.001*
Sex (F/M)	68/31	51/50	
BMI (kg/m2)	26.45 ± 4.01	26.85 ± 21.35	0.870
Pain duration (Month)			
3–12 Month	50 (50.5)		
>12 Months	49 (49.5)		
Pain severity (VAS)	4.72 ± 4.96		
Disability (ODI)	25.3 ± 14.05		
Alexithymia (TAS)	56.41 ± 11.36		
QoL (SF-36)			
Physical function	70.17 ± 23.07		
Role physical	46.71 ± 40.82		
Role emotional	52.18 ± 37.84		
Vitality	51.06 ± 20.41		
Mental health	62.45 ± 17.71		
Social functioning	69.65 ± 24.3		
Bodily pain	52.12 ± 23.36		
General health	51.61 ± 20.53		

*P < 0.05, BMI: Body masss index, F: Female, M: Male, VAS: Visual analog scale, ODI: Oswestry disability index, TAS: Toronto aleksitymia scale, QoL: Quality of life.

### 3.2. Reliability

The internal consistency of BARQ-T’s 4 factors was found between 0.883 to 0.967 Cronbach’s alpha value for CLBP suggesting good-excellent internal consistency. The internal consistency of BARQ-T’s 4 factors was found between 0.649 and 0.891 Cronbach’s alpha value suggesting acceptable-good internal consistency. Function, feelings, awareness factors were found to have excellent test-retest reliability (ICC = 0.903, ICC = 0.967, ICC = 0.927; respectively) and mood factor (ICC = 0.897) was found to have good test-retest reliability. Mean scores of the BARQ-T for test-retest, Cronbach’s alpha values and the ICC determined for test-retest reliability with 95% confidence interval were shown in Table 2. According to Table 2, patients with CLBP had lower body awareness ratio than HC.

**Table 2:** Cronbach’s alpha values, test-rest and intraclass correlation coefficient values according to BARQ-T’s factors.

BARQ-Tfactors	Cronbach’s alpha aalues		Test/RetestX ± SD			ICC (%95 CI lower-higher)	
	CLBP	HC	CLBP	HC	P	CLBP	HC
Funtion	0.904	0.649	22.51 ± 8.51 / 24.39 ± 9.36	29.81 ± 6.37 / 31.09 ± 5.71	0.00*	0.903 (0.830–0.944)	0.654 (0.387–0.804)
Mood	0.883	0.825	19.09 ± 9.04 /19 ± 8.22	22.31 ± 9.28 / 23.11 ± 10.0	0.01*	0.879 (0.789–0.931)	0.822 (0.688–0.899)
Feelings	0.967	0.743	31.88 ± 7.72 / 31.31 ± 8.62	35.03 ± 5.66 / 35.47 ± 5.66	0.001*	0.967 (0.943–0.981)	0.743 (0.547–0.854)
Awareness	0.926	0.819	25.82 ± 8.10 / 25.21 ± 8.89	28.82 ± 6.75 / 29.3 ± 6.77	0.016*	0.927 (0.872–0.958)	0.820 (0.683–0.898)

*P < 0.05, CLBP: Chronic low back pain, HC: Healthy control; X: Mean; SD: Standard deviation, ICC: Intraclass correlation coefficient, CI: Confidence interval.

### 3.3. Validity

The correlation between VAS, ODI, TAS, SF 36 and BARQ-T were assessed for construct validity study of the scale. The results of the correlations were given in Table 3. There was a negative, moderate, statistically significant correlation between ODI and function and awareness factors of BARQ-T (r1 = –0.340, r2 = –0.301; P < 0.001). There was a negative weak to moderate, statistically significant correlation between TAS and function, mood, feeling and awareness factors of BARQ-T (r1 = –0.271, r2 = –0.336, r3 = –0.394, r4 = –0.216; P < 0.05). There was no correlation between pain severity and factors of BARQ-T (P > 0.05). A positive weak to moderate statistically significant correlation were found between SF-36 subparameters and mood, feeling and function factors of BARQ-T (Table 3). For discriminative validity, the AUC was found 0.74 for function factor; 0.60 for mood factor, 0.63 for feeling factor and 0.59 for awareness factor. The results of discrimination between HC and patients with CLBP presented as 95% CI (Table 4).

**Table 3:** TThe correlations between factors of the BARQ-T and questionnaires/scales.

Questionnaires/Scales	BARQ-T factors							
	Function		Mood		Feelings		Awareness	
	r	(P)	r	(P)	r	(P)	r	(P)
Pain severity (VAS)	–0.083	0.415	0.171	0.091	0.058	0.566	0.076	0.457
Disability(ODI)	–0.340	0.001**	–0.092	0.365	–0.106	0.295	–0.301	0.003**
Alexithymia (TAS)	–0.271	0.007**	–0.336	0.001*	–0.394	0.000**	–0.216	0.032*
QoL (SF-36)								
Physical functioning	0.452	0.000**	0.176	0.082	0.220	0.029*	0.408	0.000**
Role physical	0.483	0.000*	0.169	0.094	0.246	0.014*	0.301	0.003*
Role emotional	0.411	0.000**	0.231	0.022*	0.335	0.001*	0.216	0.033*
Vitality	0.384	0.000**	0.181	0.073*	0.339	0.001**	0.147	0.150
Mental health	0.343	0.001**	0.113	0.264	0.334	0.001 **	0.197	0.052
Social functioning	0.408	0.000**	0.261	0.009**	0.341	0.001**	0.107	0.295
Bodily pain	0.434	0.000**	0.137	0.178	0.254	0.011*	0.273	0.007**
General health	0.502	0.000**	0.311	0.002**	0.382	0.000**	0.337	0.001**

*P < 0.05, **P < 0.001, VAS: Visual analog scale, ODI: Oswestry disability index, TAS: Toronto alexithymia scale, QoL: Quality of life.

**Table 4:** Discriminate ability of the 4 factors of BARQ-T, contrasting test scores of healthy persons and patients with CLBP using ROC curve analysis, reporting the confidence interval (CI).

BARQ-T	AUC	P	95% CI
Function	0.748	0.03*	0.679–0.817
Mood	0.605	0.04*	0.526–0.683
Feelings	0.632	0.04*	0.554–0.710
Awareness	0.599	0.04*	0.520–0.678

*P < 0.05, AUC: Area under curve, CI: Confidence interval.

## 4. Discussion

The current study investigated the validity and reliability of BARQ-T, which includes 4 different factors of body awareness in patients with CLBP, and the study found good-excellent Cronbach’s alpha values for patients with CLBP and acceptable-good Cronbach’s alpha values for HC and ICC values.

For test-retest validity were found to be good-excellent for patients with CLBP in all factors and acceptable-good, except for the function factor of BARQ-T in HC. Our results showed that the BARQ-T was positively associated with QoL and negatively associated with disability and alexithymia scales. The study confirmed that the BARQ-T has acceptable validation and reliability in Turkish CLBP community. The function factor demonstrated acceptable discriminate ability, and this property was not satisfactory for other factors.

Factors of BARQ-T were found higher in HC than patients with CLBP showed the deterioration of body awareness by having CLBP. According to neuro imagination studies, structural and functional alterations of cortex were found in patients with CLBP. Craig et al. reported that many of neural pathways stimulated by pain and these pathways activated some interceptive regions simultaneously [6]. Interception identified as whole afferent inputs, which affects cognition or attitude of humans in conscious or unconscious conditions. Sensations could lead to cortex level with interoceptive awareness and improvements in pain management could be achieved. CLBP occupied 30% of all chronic pain conditions. Improving interoceptive awareness should be done for validation and reliable methods in pain management. Most of the studies found negative correlations between body awareness and pain severity in patients with CLBP. Another study reported that psychological experience of pain was correlated with body awareness and it was more important than pain severity. This study also concluded that pain is a kind of behavior and the factors related to pain behavior alter the pain perception [22]. In the current study, no correlation was found between pain severity and factors of BARQ-T, but it showed that there should be other factors affecting pain behavior. It is stated as a limitation of our study not to apply depression or anxiety scales to patients with CLBP due to body awareness potentially being a psychological condition. It was reported that pain severity causes alterations in body awareness and patients developed fear avoidance beliefs and functional limitations [23]. It has been known that patients with severe disability have more deterioration in pain perception than those with mild disability [24]. Our study supported previous studies in terms of all factors of BARQ-T decreasing as disability increases. Most of the patients with CLBP believed that they tend to injure their lower back and they could not achieve any of the movements as to healthy peers [25, 26]. These thoughts cause differences in pain perception, such as feeling pain in the lower back and the body behavior changing as consequence. The authors of this study believed that disability levels increased due to pain severity changing body awareness and alterations in body awareness reflected the disability. Wand et al. reported that deteriorated body awareness associated with pain severity, pain catastrophizing, fear avoidance beliefs, and psychological conditions in patients with CLBP, should be a prior assessment to discriminate pain severity [23]. Hence, an easily applicable, simple, practical, reliable and valid Turkish version of BARQ was strongly needed in pain management and recommended to use in patients with CLBP for further studies.

Shibata et al. reported that development of chronic pain has positive correlations with patients with chronic pain and emphasized that patients who had chronic pain had severe alexithymia than those with emotional insufficiency [27]. Due to alexithymia and body awareness, which are transmitted by the same neural pathways and structures, decreases in body awareness were found to be associated with an increase in alexithymia [28]. The current study found similar results in terms of negative correlations between body awareness and alexithymia. It is not surprising that there were positive correlations between QoL and body awareness in terms of SF-36 and BARQ-T, with mutual subparameters such as mood, emotional status and physical function. Research on patients with chronic musculoskeletal pain showed increase in QoL, decrease analgesic requirement and anxiety after body awareness treatments [29].

Perception of pain in all aspects and participation of activity are mutual points of all effective treatments on chronic pain. Moreover, keeping this lifestyle decreases pain severity, is gained pain management and coping with fear avoidance [22]. Multidimensional assessments are needed to do effective treatments and provide pain management. We recommend the use of BARQ-T in terms of pain perception and pain assessment in low back pain which incidence is high in Turkish society.

## Acknowledgment

The authors would like to thank Erkan Sumer MD, for referral to participants to the physiotherapy. The authors would like to thank Corey DeLoach for revisions and clarity of writing.

## Disclaimers/Conflict of interest

The authors declare no conflict of interest. No funding has received for thisstudy.

## Informed consent

The study was approved by Non-Entrepreneurial Clinical Studies Ethical Board of Hacettepe University (Ethics Committee Registration No: GO 18/981). All participants provided informed consent in the format required by the relevant authorities and/or boards.
